# The impact of spectral filtration on image quality in micro‐CT system

**DOI:** 10.1120/jacmp.v17i1.5714

**Published:** 2016-01-08

**Authors:** Liqiang Ren, Muhammad U Ghani, Di Wu, Bin Zheng, Yong Chen, Kai Yang, Xizeng Wu, Hong Liu

**Affiliations:** ^1^ Center for Biomedical Engineering and School of Electrical and Computer Engineering, University of Oklahoma Norman OK; ^2^ Department of Radiation Oncology University of Oklahoma Health Sciences Center Oklahoma City OK; ^3^ Department of Radiology Massachusetts General Hospital Boston MA; ^4^ Department of Radiology University of Alabama at Birmingham Birmingham AL USA

**Keywords:** spectral filtration, image quality, microcomputed tomography (micro‐CT), contrast‐to‐noise ratio (CNR), tissue characteristic

## Abstract

This paper aims to evaluate the impact of spectral filtration on image quality in a microcomputed tomography (micro‐CT) system. A mouse phantom comprising 11 rods for modeling lung, muscle, adipose, and bones was scanned with 17 s and 2 min, respectively. The current (μA) for each scan was adjusted to achieve identical entrance exposure to the phantom, providing a baseline for image quality evaluation. For each region of interest (ROI) within specific composition, CT number variations, noise levels, and contrast‐to‐noise ratios (CNRs) were evaluated from the reconstructed images. CT number variations and CNRs for bone with high density, muscle, and adipose were compared with theoretical predictions. The results show that the impact of spectral filtration on image quality indicators, such as CNR in a micro‐CT system, is significantly associated with tissue characteristics. The findings may provide useful references for optimizing the scanning parameters of general micro‐CT systems in future imaging applications.

PACS numbers: 87.57.C‐, 87.57.Q‐, 87.64.kd

## I. INTRODUCTION

As a scaled‐down computed tomography (CT) imaging modality, micro‐CT was initially developed for industrial imaging applications and then for small animal imaging, but it recently has proven to be a useful tool in various clinical medical applications such as the assessments of the three‐dimensional (3D) bone microstructure and bone mineral density (BMD) performed on human legs or on bone biopsy specimens, the study of microvasculature anatomy, and the imaging of tumor perfusion and angiogenesis.^(1)^ Micro‐CT is capable of providing 3D imaging at high resolution on the order up to 10 μm, enhancing the way of noninvasively and precisely visualizing the microarchitecture inside the object.^2^, ^3^, ^4^, ^5^, ^6^, ^7^

Characterizing and optimizing the imaging performance of micro‐CT have been widely investigated in the past decades with simulation and experimental methods. Primary studies include the determination of the radiation dose levels using Monte Carlo procedure or ion‐chamber dosimeter, the assessment of system resolution in slanted edge and plane spread function methods, the investigation of noise propagation and reduction, and other evaluations for imaging quality assurance such as geometric accuracy, linearity, CT number variation, as well as uniformity.^8^, ^9^, ^10^, ^11^ Inherent and additional filtrations are generally applied in micro‐CT to eliminate low‐energy photons that make less contribution to image quality, but tend to increase the absorbed radiation dose.^8^, ^9^, ^11^ However, their effectiveness and influence on CT number variation, noise level, and image quality have not been fully characterized to date. Though the influence of spectral filtration on patient dose, as well as image quality, has been studied on a conventional CT scanner, the imaging parameters and scenario are different as in a micro‐CT system.^(12)^ Therefore, it is necessary and meaningful to perform a comprehensive investigation of the impact of spectral filtrations on image quality in a micro‐CT system.

In this study, with the use of two imaging phantoms, we investigated the impact of spectral filtrations, including aluminum (Al), copper (Cu) and their combinations, on CT number variation, noise level, and image quality indicators such as contrast‐to‐noise ratio (CNR). Based on the evaluations of CNRs for soft tissue, muscle, and bone with low density, we further analyzed the influence of spectral filtrations on low‐contrast resolution, an important estimator in micro‐CT imaging.^13^, ^14^ Our overall objective is to assess the feasibility of developing and applying a new evaluation tool that can provide useful evaluation results or references for selecting the optimal scanning parameters in micro‐CT systems in future imaging applications.

## II. MATERIALS AND METHODS

### A. Micro‐CT scanner

A rotating gantry based micro‐CT system (Quantum FX, Perkin Elmer, Waltham, MA) was used in this study. The major components of the scanner consist of a polychromatic X‐ray source, of which the anode is constructed from tungsten (W), and a flat panel X‐ray imaging detector positioned opposite to each other on the gantry with a fixed source‐to‐detector distance of 265 mm. The X‐ray source is capable of providing target voltages from 30 to 90 kVp, with a current range from 20 to 200 μA. Inherent filtrations with the purpose of reducing the fluence of low‐energy photons include a 150 μm thick output window of beryllium (Be), 100 μm Al, and 60 μm Cu. Two modes are available for this scanner, live mode and CT scan mode. The live mode is used to view the subjects in real time with/without rotating the gantry, and the exposure lasts for up to 150 s, providing the possibility of spectral measurement. In contrast, various scan settings of standard scan and fine scan are provided in the CT scan mode, depending on the selected field of view (FOV).

### B. Spectral measurement and evaluation

**Figure 1:**
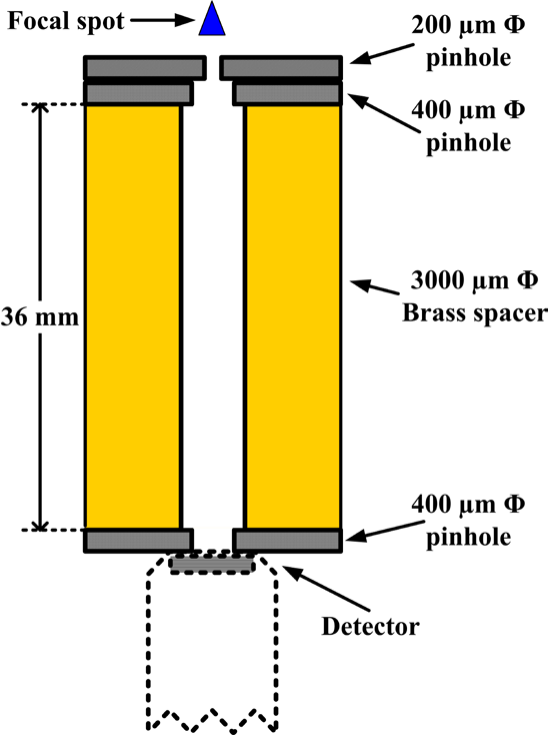
Configuration of spectrometer collimator used for covering the CdTe detector while limiting the X‐ray photon rate to an acceptable level.

X‐ray spectra were measured using a cadmium telluride (CdTe) spectrometer (XR‐100T‐CdTe, Amptek, Bedford, MA). The spectrometer performing peak efficiency at 10−100 keV includes an X‐ray and gamma ray detector, a preamplifier, and a cooler system using 3×3×1 mm CdTe diode detector mounted on a two‐stage thermoelectric cooler. During the spectral measurement, the spectrometer was positioned with its axis perpendicular to the imaging detector. Considering the extremely limited space inside the gantry (i.e., extremely short distance between X‐ray source and spectrometer collimator), three pinholes with small diameters and 2 mm in thickness were applied with a brass spacer (36 mm in length and 3000 μm in diameter) separating them to cover the CdTe detector, in an effort to limit the photon rate to an acceptable level and prevent spectral distortions due to pileup. As shown in Fig. 1, two pinholes on top of the spacer had 200 μm and 400 μm diameters, and the one below had 400 μm diameter. Since the received photon rate is very sensitive to the position of the spectrometer due to the collimator restriction, a good alignment of the spectrometer–pinhole assembly to the central ray from the X‐ray focal spot, therefore, becomes essential in order to achieve an accurate spectral measurement. The best position was determined through repeatedly moving the spectrometer until finding a “sweet spot” with the highest photon rate and proper spectral shape.^(15)^

The spectra were measured with inherent filtration and various additional spectral filtrations in the live mode under the condition of 90 kVp, 200 μA and 150 s. The additional spectral filtrations included Al, Cu, and their combinations: 0.5 mmAl, 1.0 mmAl, 1.5 mmAl, 2.0 mmAl, 2.5 mmAl, 3.0 mmAl, 3.5 mmAl, 4.0 mmAl, 0.2 mmCu, and 0.2 mmCu+2.5 mmAl. For each measuring condition, three spectra were acquired and averaged to reduce the stochastic noise.

To evaluate the spectral measurement accuracy, energy resolution (R) of the characteristic peak $K_{\alpha 1}$ was calculated using Eq. (1), where $\Delta E_{\text{FWHM}}$ is the full width in energy unit at half maximum, and $E_{C}$ is the central energy (59.32 keV) of $K_{\alpha 1}$:^(16)^

(1) $$R=\frac{\Delta E_{FWHM}}{E_{C}}\times 100\%$$

The beam quality generally defined as the overall ability of an X‐ray beam to penetrate an object was evaluated by calculating the mean energy and half value layer (HVL), which provide the criteria for beam quality improvements with increasingly additional filtrations. The mean energy was directly calculated from the measured spectra using Eq. (2), where ME is the mean energy, E is the energy for each channel, and N(E) is the photon count in the channel with energy E:

(2) $$ME=\frac{\sum_{E=0keV}^{90keV}E\cdot N(E)}{\sum_{E=0keV}^{90keV}N(E)}$$

In contrast, the HVL was measured using sheets of Al (type‐1100) and a recently calibrated ion chamber.^(16)^ During the HVL measurement, the ion chamber was flatly placed inside the gantry facing upward to the X‐ray focal spot and the sensitive as well as the geometric center of the ion chamber coincided with the isocenter of the rotating gantry.

### C. Imaging phantoms and scan protocol

**Figure 2:**
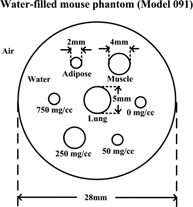
Schematic of the water‐filled mouse phantom; the length is 30 mm for the rod representing lung, while 32 mm for other rods.

Two imaging phantoms were used in this study, a uniform water phantom with a diameter of 28 mm for measuring the CT number uniformity, and a water‐filled mouse phantom (Model 091, CIRS, Norfolk, VA) for evaluating the impact of the additional filtrations on the image quality. Note that these two phantoms were filled with deionized (DI) water to avoid any unnecessary imaging uncertainties. The mouse phantom consists of 11 rods of varying mineral loading and dimension in a water‐tight, polycarbonate housing which is very durable and resistant to many chemicals. Seven rods representing different compositions (i.e., lung, muscle, adipose, and bone with various densities of 0, 50, 250, and 750 mg/cc) within mammals were selected. The varied bone densities are realized through blending hydroxyapatite (HA), the principal constituent of teeth and bones within mammals, in a soft‐tissue equivalent, polymer background. The cross section containing the rods of interest is shown in Fig. 2 with more detailed dimensions.

Under each spectral filtration condition, the water phantom and the mouse phantom were scanned with 40 mm FOV and 90 kVp, but different currents adjusted from 60μA to 200μA to provide the same entrance exposure to the object as a baseline for CT number uniformity and image quality comparison. The entrance exposure was measured using the same ion chamber and in the same way as in the HVL measurements. Through adjusting the current while keeping the same exposure time (e.g., 12 s), similar exposure levels were achieved, and the corresponding currents were determined for different scan conditions. Note that the process of acquiring equivalent entrance exposure was performed under the micro‐CT live mode, but the determined currents were applicable to the scan mode due to the constant source‐to‐object distance. Then, two CT scans, a standard scan of 17 s and a fine scan of 2 min, were performed on both imaging phantoms under each scan protocol.

### D. CT number uniformity measurement and image quality assessment

**Figure 3:**
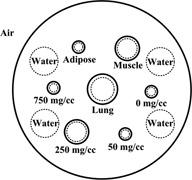
Eleven ROIs defined for water, lung, muscle, adipose, and bones.

With various spectral filtrations, the reconstructed images acquired from the water phantom were analyzed to compare the CT number uniformity, while those from the mouse phantom were to compare the CT numbers, noise levels, and CNRs of regions of interest (ROIs) for different materials within the mouse phantom. Since the source spectrum was significantly modified with the interaction of spectral filtrations, CT number calibration was performed with two specifically selected ROIs, one for air and the other for water, before the image acquisition under each scanning setting. The averaged CT number for the air ROI was calibrated as −1000, while 0 for the water ROI. Then, eleven circular ROIs with different diameters were defined, four for water as well as bones, and one for lung, muscle, adipose each, as in Fig. 3. The mean value within each ROI in the current slice was regarded as the CT number, ExperCTk for that corresponding material k of interest.

In addition, the CT number for each material could be theoretically predicted using Eq. (3) with the *a priori* knowledge of mean energy and linear attenuation coefficients (LACs): where k indicates the material of interest (e.g., lung, muscle, adipose, bones, and water) and ${\text{Theor}\_\text{CT}}_{k}$ is the theoretical CT number for material k, and $\mu_{k,\text{ME}}$ and $\mu_{\text{bg},\text{ME}}$ represent the LACs under the mean energy for material and background of water.^(16)^

(3) $$Theor\_CT_{k}=\frac{\mu_{k,ME}-\mu_{bg,ME}}{\mu_{bg,ME}}\times 100$$

In order to minimize the background information such as ring artifacts, beam hardening artifacts, streaking artifacts, and cone‐beam artifacts, two sequential acquisitions of the image were acquired, and the noise was calculated from the subtraction of one acquisition from the other. Specifically, the noise level $\left(N_{\text{bg}}\right)$ was determined through dividing the standard deviation (SD) in the four water ROIs on the subtraction image by a factor of $\sqrt{2}$, as shown in Eq. (4):^(17)^

(4) $$N_{bg}=SD/\sqrt{2}$$

Then, the experimental and theoretical CNRs of each material of interest were calculated using (5), (6). In Eq. (6), the relationship between theoretical CNR and LAC under mean energy for each material was established through substituting Eq. (3) and considering ${\text{Theor}\_\text{CT}}_{\text{bg}}=0$.

(5) $$Exper\_CNR_{k}=\frac{Exper\_CT_{k}-Exper\_CT_{bg}}{N_{bg}}$$

(6) $$\begin{array}{ccc} Theor\_CNR & = & \frac{Theor\_CT_{k}-Theor\_CT_{bg}}{N_{bg}}\\ & = & \frac{\frac{\mu k,ME-\mu bg,ME}{\mu bg,ME}\times 1000}{N_{bg}} \end{array}$$

The standard derivations ($\sigma_{\text{Exper}\_\text{CN}\;\text{Rk}}$ and $\sigma_{\text{Theor}\_\text{CN}\;\text{Rk}}$) of experimental and theoretical CNRs were calculated using (7), (8), where $\sigma_{\text{Exper}\_\text{CTk}}$, $\sigma_{\text{Exper}\_\text{CTbg}}$, $\sigma_{\text{Nbg}}$, $\sigma_{\mu k,\text{ME}}$ and $\sigma_{\mu \text{bg},\text{ME}}$ were the standard derivations of ExperCTk, ExperCTbg, Nbg, μk,ME and μbg, ME either acquired experimentally (multiple measurements) or theoretically (e.g., curve fitting).

(7) $$\sigma Exper\_CNR_{k}=\frac{1}{N_{bg}}\sqrt{\sigma_{Exper\_CT_{k}}^{2}+\sigma_{Exper\_CT_{bg}}^{2}+\frac{{\left(Exper\_CT_{k}-Exper\_CT_{bg}\right)}^{2}\sigma_{N_{bg}}^{2}}{N_{bg}^{2}}}$$

(8) $$\sigma Theor\_CNR_{k}=\frac{1000}{\mu_{bg}{\;}_{,ME}N_{bg}}\sqrt{\sigma_{\mu k,ME}^{2}+\frac{\mu_{k,ME}^{2}\sigma_{\mu bg,ME}^{2}}{\mu_{bg,ME}^{2}}+\frac{{\left(\mu k,ME-\mu bg,ME\right)}^{2}\sigma_{N_{bg}}^{2}}{N_{bg}^{2}}}$$

In summary, 50 consecutive slices in the reconstructed images were selected for experimental assessments of CT number, noise level, and CNRs in each test.

## III. RESULTS

### A. Spectra evaluation and current determinations

**Figure 4:**
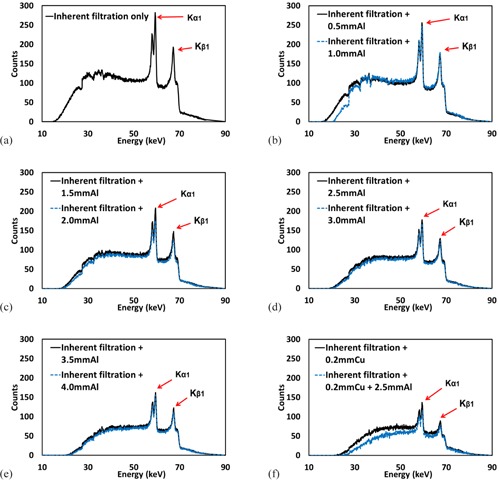
Measured spectra with (a) inherent filtration only and (b)–(f) various additional filtrations.

As shown in the second column of Table 1, all spectra were acquired with acceptable photon rates less than 2000 photons per second (p/s) corresponding to a photon flux of about $222\;\text{photons per second per}\;{\text{mm}}^{2}\;(p/s/{\text{mm}}^{2})$ so as to avoid the pileup phenomenon. For each spectrum, the channel‐to‐energy calibration were performed using two characteristic peaks from K line of W, $K_{\alpha 1}$, and $K_{\beta 1}$. Then, energy resolution and mean energy were derived using (1), (2) from the measured spectra and listed in the third and fourth column of Table 1. Due to the restriction of the received photon rate, high energy resolutions ranging from 1.46% to 2.05% were achieved. According to the calculation of mean energy and the measurement of HVL (fifth column), the beam quality was improved with increasingly additional filtrations. In the sixth column, the current (μA) for each filtration was determined to provide identical entrance exposure, as a baseline for image quality comparison with various spectral filtrations. Each entrance exposure lasted for 12 s; the values in the last column being the average of three repeated measurements, with a maximum relative deviation below 2.1%. All measured spectra are demonstrated in Fig. 4, indicating improved beam qualities with increasingly additional filtrations.

**Table 1:** Spectral evaluations with respect to photon rate, energy resolution, and mean energy, HVL measurement in terms of the thickness of Al, and current determination providing identical entrance exposure; all these data were calculated or measured at 90 kVp.

*Additional Filtration (mm)*	*Photon Rate (p/s)*	*Energy Resolution (R: %)*	*Mean Energy (keV)*	*HVL (mmAl)*	*Current (μA)*	*Entrance Exposure (12s: mR)*
None	1930±55	1.65±0.15	46.68±0.11	3.48	63	474±5.0
0.5Al	1723±25	1.65±0.15	47.53±0.17	3.69	72	470±7.3
1.0Al	1552±29	1.60±0.18	47.99±0.07	3.83	80	480±6.8
1.5Al	1397±14	1.46±0.18	48.70±0.04	4.04	87	474±5.4
2.0Al	1272±32	1.89±0.00	49.01±0.17	4.34	98	471±9.9
2.5Al	1198±10	1.84±0.35	49.79±0.04	4.72	107	480±1.8
3.0Al	1102±12	1.65±0.15	50.15±0.08	4.94	118	472±2.6
3.5Al	1051±13	1.94±0.31	50.73±0.06	5.17	129	474±8.8
4.0Al	971±7	1.65±0.15	51.09±0.09	5.41	140	477±4.5
0.2Cu	938±11	1.97±0.11	51.16±0.10	5.45	144	472±8.3
0.2Cu+2.5Al	724±5	2.05±0.11	52.94±0.04	6.81	200	474±4.0

### B. CT number uniformity and image quality analysis

**Figure 5:**
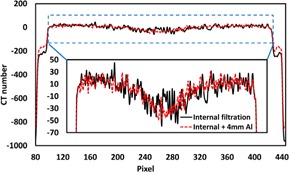
CT number profiles using a uniform water phantom, one with internal filtration only and the other with additional 4 mm Al filtration.

The CT number uniformities were compared by plotting the CT number profile of the central line in each acquired image from the water phantom. The comparison with an internal filtration only and with an additional 4 mm Al filtration is presented in Fig. 5, showing that both uniformities are acceptable. This insignificant improvement may be because that the object used in this micro‐CT imaging system is relatively small, and the internal filtration of Al and Cu has already removed most of the X‐ray photons with low energies which are the major contributor to the beam hardening effect.

Five compositions (i.e., water, air, muscle, adipose, and bone with high density) were selected for the theoretical calculations and predications of CT number and CNR. The LACs of these compositions were obtained from the National Institute of Standards and Technology (NIST, Gaithersburg, MD), but only with discrete energies (e.g., 10 keV, 20 keV).^(18)^ In order to acquire the LAC data under the mean energies (46.68–52.94 keV) when different spectral filtrations were utilized, a second‐order polynomial fitting method was applied on the available data at the energies of 40 keV, 50 keV, and 60 keV for each composition, and the fitting results are listed in Table 2. Corresponding CT number calculated theoretically using Eq. (3) is presented, as well. It is not surprising that the LAC for each composition is decreased with the improved beam quality due to the increased mean energy, as well as the stronger penetrability. Also, the configuration is consistent for all filtrations because the CT number in air and water are very similar or equal, while for adipose, muscle, and bone, the absolute CT numbers are monotonically decreased with increasingly additional filtrations.

**Figure 6:**
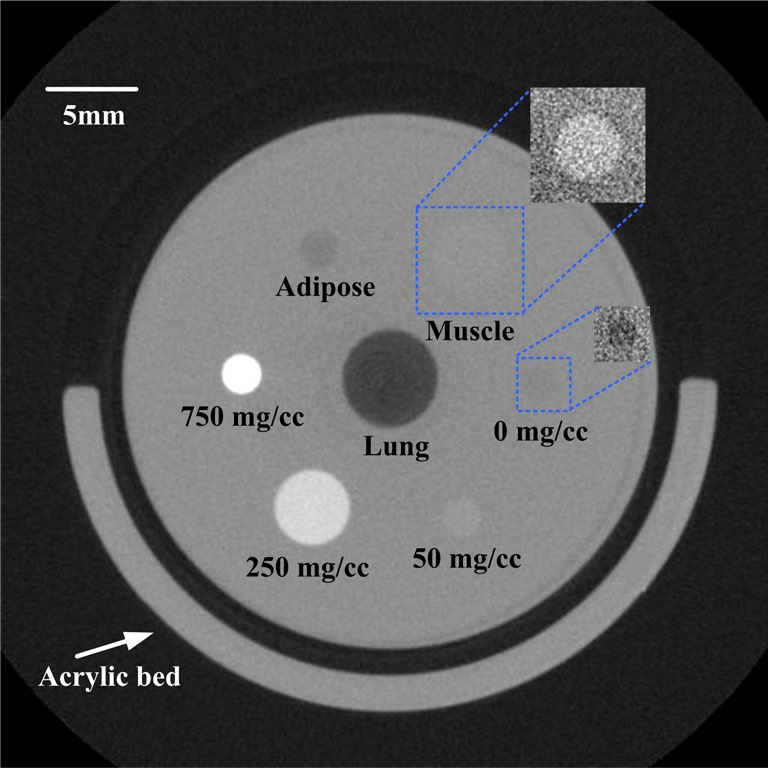
Reconstructed image sample of mouse phantom; the gray level values are scaled for muscle and 0 mg/cc.

The CT numbers acquired by experiments for water, lung, adipose, muscle, and bones are shown in 3, 4 under the scan condition of 17 s and 2 min, respectively, and a reconstructed image sample of mouse phantom is shown in Fig. 6. The results of CT number determination agree with the theoretical calculations with regard to water, adipose, and bone with high density. The CT number variations of muscle and bone with low density (e.g., 50 mg/cc) are not obvious, because their LACs are quite similar as that of water and, therefore, the results are prone to be affected by the imaging noise. Since most part of the lung is composed by air, the lung CT number shows a variation tendency towards to −1000 as that of air rather than to 0.

The results determined by Eq. (4) indicate a reduction of noise with additional spectral filtrations under both standard and fine scan, as shown in Table 5. The trends seen in the table may be caused by improved beam quality and narrower spectra with filtration. Also, the reduction of noise provides a potential to achieve improved CNR for certain materials based on the tissue characteristic.

Table 6 summarizes the theoretical CNRs and the corresponding standard derivations derived from (6), (8) for muscle, adipose, and bone $\left(1.92\;g/{\text{cm}}^{3}\right)$, respectively, while the experimental CNRs and their standard derivations directly determined using (5), (7) for lung, muscle, adipose, and bones (0−750 mg/cc) are summarized in 7, 8. The theoretical and experimental results have high association. Both show that the CNR for bone with higher density (e.g., $1.92\;g/{\text{cm}}^{3}$, 750 mg/cc, and 250 mg/cc) decreases with additional spectral filtrations. Similar phenomenon is also observed in soft tissue, such as adipose and bone with a density of 0 mg/cc. However, opposite situations occur in lung, muscle, and bone with lower density (50 mg/cc). Thus, the imaging quality indicator in terms of CNR significantly depends on the tissue characteristic, which indicates that experimental data are needed to provide useful references or guidelines for the utilization of additional spectral filtrations in future micro‐CT imaging applications.

**Table 2:** Determination of LACs $\left({\text{cm}}^{-1}\right)$ and CT numbers (Hounsfield unit (HU)) using a second‐order polynomial fitting and Eq. (3).

*Additional Filtration (mm)*	*Mean Energy (keV)*	*Water* $\left(1.00\;g/{\text{cm}}^{3}\right)$		*Air* $\left(0.001205\;g/{\text{cm}}^{3}\right)$		*Adipose* $\left(0.95\;g/{\text{cm}}^{3}\right)$		*Muscle* $\left(1.05\;g/{\text{cm}}^{3}\right)$		*Bone* $\left(1.92\;g/{\text{cm}}^{3}\right)$	
		*LAC*	*CT*	*LAC*	*CT*	*LAC*	*CT*	*LAC*	*CT*	*LAC*	*CT*
None	46.68	0.238	0	2.64E−1	−998.892	0.209	−123.304	0.250	47.989	0.940	2944.062
0.5Al	47.53	0.235	0	2.60E−1	−998.893	0.207	−120.025	0.246	47.660	0.905	2848.796
1.0A1	47.99	0.234	0	2.59E−1	−998.893	0.206	−118.293	0.245	47.486	0.887	2798.434
1.5Al	48.70	0.231	0	2.56E−1	−998.894	0.204	−115.685	0.242	47.223	0.860	2722.482
2.0Al	49.01	0.230	0	2.54E−1	−998.894	0.204	−114.571	0.241	47.110	0.849	2690.035
2.5Al	49.49	0.228	0	2.51E−1	−998.895	0.202	−111.844	0.238	46.834	0.822	2610.437
3.0Al	50.15	0.226	0	2.50E−1	−998.896	0.201	−110.622	0.237	46.709	0.809	2574.734
3.5Al	50.73	0.225	0	2.48E−1	−998.896	0.200	−108.706	0.235	46.513	0.791	2518.663
4.0Al	51.09	0.224	0	2.47E−1	−998.896	0.200	−107.550	0.234	46.394	0.779	2484.795
0.2Cu	51.16	0.223	0	2.47E−1	−998.897	0.199	−107.328	0.234	46.372	0.777	2478.295
0.2Cu+2.5Al	52.94	0.219	0	2.41E−1	−998.898	0.196	−102.045	0.229	45.824	0.726	2322.955

**Table 3:** CT numbers (HU) determined by experiments under a standard scan of 17 s, the values below being the average derived from 50 consecutive slices in the reconstructed image. (Note: the standard deviation value is not associated with the CT number in a given slice, but is derived as the variation of CT numbers from 50 consecutive slices in the reconstructed images for each material.)

*Additional Filtration (mm)*	*Water*	*Lung*	*Adipose*	*Muscle*	*Bone 0 mg/cc*	*Bone 50 mg/cc*	*Bone 250 mg/cc*	*Bone 750 mg/cc*
None	−1.584±1.032	−631.432±108.613	−113.181±109.614	33.271±107.614	−42.041±107.383	82.472±108.382	591.763±111.523	1543.741±111.532
0.5Al	−4.495±1.042	−642.102±106.194	−113.952±105.102	32.771±104.603	−43.883±105.431	76.683±105.712	577.132±108.151	1501.132±107.215
1.0Al	−1.768±0.982	−645.553±104.812	−119.803±101.974	37.184±102.762	−33.924±101.572	83.321±102.541	568.721±106.052	1483.112±105.045
1.5Al	−2.172±0.990	−647.632±104.632	−105.421±102.402	37.973±102.302	−28.841±101.762	84.084±103.123	561.462±105.189	1453.204±103.741
2.0Al	−0.864±1.023	−647.652±102.602	−104.472±102.072	37.372±101.612	−24.824±100.863	87.152±97.681	552.923±103.794	1418.161±107.314
2.5Al	1.037±1.024	−649.341±101.182	−102.871±97.364	39.324±98.362	−19.721±96.614	85.905±98.734	544.462±100.893	1401.784±101.052
3.0Al	0.910±0.988	−650.512±101.193	−93.814±100.253	39.903±98.712	−21.342±97.264	87.922±100.041	534.581±101.052	1371.182±105.142
3.5Al	−0.505±0.986	−648.403±102.042	−99.403±99.962	35.432±99.703	−18.352±99.662	83.631±99.431	524.612±101.812	1335.183±103.653
4.0Al	−0.841±1.001	−649.451±101.321	−98.743±99.293	37.871±99.173	−17.114±98.691	87.451±98.671	517.254±100.863	1326.264±97.426
0.2Cu	2.489±2.032	−656.023±103.562	−90.763±100.593	40.332±99.881	−1.813±98.762	92.312±99.441	512.413±100.774	1284.083±114.835
0.2Cu+2.5Al	1.000±1.965	−661.301±100.762	−91.132±96.582	39.713±96.582	−2.652±95.743	91.123±96.812	484.612±97.962	1182.583±118.071

**Table 4:** CT numbers (HU) determined by experiments under a fine scan of 2 min, the values below being the average derived from 50 consecutive slices in the reconstructed image. (Note: the standard deviation value is not associated with the CT number in a given slice, but is derived as the variation of CT numbers from fifty consecutive slices in the reconstructed images for each material.)

*Additional Filtration (mm)*	*Water*	*Lung*	*Adipose*	*Muscle*	*Bone 0 mg/cc*	*Bone 50 mg/cc*	*Bone 250 mg/cc*	*Bone 750 mg/cc*
None	−8.588±1.001	−642.721±48.174	−124.842±41.213	31.338±41.214	−48.291±40.112	81.352±40.762	588.091±43.662	1628.621±41.832
0.5Al	−8.565±0.985	−651.594±47.123	−121.341±38.844	29.261±38.194	−45.861±37.632	81.659±39.253	581.069±42.291	1602.741±40.925
1.0Al	−6.964±1.012	−651.162±47.043	−117.082±39.001	31.232±39.112	−39.737±38.924	84.992±39.371	572.038±42.382	1572.101±40.412
1.5Al	−8.016±1.032	−652.902±47.021	−115.743±38.384	29.663±39.532	−36.921±39.333	84.403±39.443	560.463±41.942	1536.771±40.325
2.0Al	−6.721±0.974	−651.812±46.072	−113.253±39.473	32.112±38.971	−32.718±38.363	85.214±39.162	551.593±41.682	1501.982±45.565
2.5Al	−4.793±0.995	−651.572±46.012	−109.274±38.593	32.663±38.523	−29.129±37.701	86.804±38.001	544.274±40.914	1481.413±37.864
3.0Al	−4.582±0.985	−653.402±45.232	−109.650±38.614	32.001±38.001	−26.314±37.662	89.342±37.863	535.663±40.871	1447.834±43.505
3.5Al	−4.780±1.015	−654.602±45.241	−107.251±38.623	33.602±37.612	−20.749±37.802	90.403±37.871	526.824±40.302	1420.723±43.065
4.0Al	−4.201±1.014	−655.162±45.583	−106.552±38.261	34.592±37.301	−18.833±37.332	89.381±37.624	519.687±40.241	1396.775±46.562
0.2Cu	−0.392±2.021	−659.331±49.421	−102.193±38.831	38.102±38.832	−8.041±37.615	96.923±38.221	514.692±40.704	1371.889±54.912
0.2Cu+2.5Al	−2.929±2.012	−662.041±49.672	−98.001±38.614	36.003±38.492	−6.962±37.703	91.932±37.473	481.197±39.562	1268.388±57.803

**Table 5:** Noise evaluation with various spectral filtrations under the condition of standard scan of 17 s and fine scan of 2 min.

*Additional filtration (mm)*	*17 s*	*2 min*
None	64.987±5.718	24.111±0.829
0.5Al	64.263±5.959	23.710±0.852
1.0Al	63.534±5.585	23.424±0.712
1.5Al	63.056±5.764	23.294±0.896
2.0Al	62.571±5.583	23.120±0.746
2.5Al	61.768±5.105	22.844±0.723
3.0Al	60.900±5.591	22.737±0.808
3.5Al	60.227±5.302	22.643±0.713
4.0Al	59.907±5.478	22.453±0.779
0.2Cu	59.542±5.443	22.434±0.681
0.2Cu+2.5Al	57.924±5.091	22.322±0.725

**Table 6:** Theoretical CNRs for bone, adipose, and muscle with additional filtrations.

*Additional Filtration (mm)*	*Bone* $\left(1.92g/{\text{cm}}^{3}\right)$		*Adipose*		*Muscle*	
	*17 s*	*2 min*	*17 s*	*2 min*	*17 s*	*2 min*
None	45.303±3.993	122.102±4.205	−1.897±0.165	−5.114±0.174	0.738±0.068	1.990±0.072
0.5Al	44.330±4.114	120.152±4.322	−1.868±0.172	−5.062±0.181	0.742±0.068	2.010±0.071
1.0Al	44.046±3.861	119.470±3.622	−1.862±0.166	−5.050±0.155	0.747±0.065	2.027±0.061
1.5Al	43.175±3.947	116.873±4.498	−1.835±0.169	−4.966±0.193	0.749±0.069	2.027±0.079
2.0Al	42.991±3.837	116.349±3.757	−1.831±0.161	−4.955±0.158	0.753±0.068	2.038±0.067
2.5Al	42.262±3.486	114.271±3.610	−1.811±0.153	−4.896±0.158	0.758±0.059	2.050±0.061
3.0Al	42.278±3.889	113.238±4.030	−1.816±0.167	−4.865±0.173	0.767±0.073	2.054±0.076
3.5Al	41.819±3.677	111.231±3.501	−1.805±0.162	−4.801±0.155	0.772±0.065	2.054±0.062
4.0Al	41.477±3.782	110.666±3.826	−1.795±0.164	−4.790±0.165	0.774±0.068	2.066±0.069
0.2Cu	41.623±3.814	110.469±3.359	−1.803±0.165	−4.784±0.146	0.779±0.076	2.067±0.067
0.2Cu+2.Al	40.104±3.513	104.066±3.369	−1.762±0.159	−4.571±0.153	0.791±0.069	2.053±0.066

**Table 7:** Experimental CNRs with standard scan of 17 s.

*Additional Filtration (mm)*	*Bone (750 mg/cc)*	*Bone (250 mg/cc)*	*Bone (50 mg/cc)*	*Bone (0 mg/cc)*	*Adipose*	*Muscle*	*Lung*
None	23.779±2.706	9.130±1.894	1.293±1.672	−0.623±1.653	−1.717±1.693	0.536±1.657	−9.692±1.876
0.5Al	23.429±2.739	9.051±1.881	1.263±1.649	−0.613±1.642	−1.703±1.643	0.580±1.629	−9.922±1.891
1.0Al	23.371±2.637	8.979±1.846	1.339±1.618	−0.506±1.599	−1.700±1.612	0.613±1.618	−10.132±1.875
1.5Al	23.081±2.675	8.939±1.858	1.368±1.640	−0.423±1.614	−1.637±1.631	0.637±1.623	−10.236±1.905
2.0Al	22.678±2.652	8.850±1.837	1.407±1.566	−0.383±1.612	−1.656±1.638	0.611±1.609	−10.337±1.881
2.5Al	22.678±2.488	8.798±1.788	1.374±1.602	−0.336±1.564	−1.682±1.582	0.620±1.593	−10.529±1.855
3.0Al	22.500±2.692	8.763±1.844	1.429±1.648	−0.365±1.597	−1.555±1.652	0.640±1.622	−10.697±1.930
3.5Al	22.177±2.603	8.719±1.857	1.397±1.655	−0.296±1.655	−1.642±1.666	0.597±1.656	−10.758±1.941
4.0Al	22.153±2.598	8.648±1.860	1.474±1.653	−0.272±1.653	−1.634±1.664	0.646±1.656	−10.827±1.960
0.2Cu	21.524±2.755	8.564±1.865	1.509±1.676	−0.072±1.659	−1.566±1.695	0.636±1.678	−11.060±2.012
0.2Cu+2.5Al	20.399±2.715	8.349±1.844	1.556±1.677	−0.063±1.653	−1.591±1.700	0.668±1.668	−11.434±2.009

**Table 8:** Experimental CNRs with fine scan of 2 min.

*Additional Filtration (mm)*	*Bone (750 mg/cc)*	*Bone (250 mg/cc)*	*Bone (50 mg/cc)*	*Bone (0 mg/cc)*	*Adipose*	*Muscle*	*Lung*
None	67.902±2.908	24.747±2.001	3.730±1.695	−1.647±1.664	−4.821±1.717	1.656±1.666	−26.300±2.193
0.5Al	67.959±2.991	24.869±1.995	3.805±1.661	−1.573±1.588	−4.756±1.647	1.595±1.612	−27.120±2.214
1.0Al	67.413±2.679	24.719±1.959	3.926±1.685	−1.399±1.662	−4.701±1.671	1.631±1.670	−27.502±2.175
1.5Al	66.316±3.083	24.404±2.031	3.967±1.700	−1.241±1.689	−4.624±1.657	1.617±1.698	−27.684±2.282
2.0Al	65.254±2.884	24.148±1.964	3.976±1.699	−1.124±1.660	−4.608±1.714	1.680±1.686	−27.901±2.187
2.5Al	65.058±2.643	24.035±1.946	4.009±1.668	−1.065±1.651	−4.573±1.695	1.639±1.687	−28.312±2.204
3.0Al	63.878±2.968	23.760±1.986	4.131±1.672	−0.956±1.657	−4.621±1.706	1.609±1.685	−28.535±2.233
3.5Al	62.954±2.748	23.477±1.927	4.203±1.678	−0.705±1.670	−4.525±1.712	1.695±1.662	−28.698±2.193
4.0Al	62.396±2.997	23.333±1.966	4.168±1.682	−0.652±1.663	−4.558±1.711	1.728±1.662	−28.992±2.265
0.2Cu	61.169±3.071	22.960±1.943	4.338±1.709	−0.341±1.676	−4.538±1.736	1.716±1.748	−29.372±2.376
0.2Cu+2.5Al	56.954±3.183	21.688±1.907	4.205±1.684	−0.181±1.689	−4.259±1.735	1.744±1.725	−29.527±2.423

## IV. DISCUSSION

Since certain bone disorders may be directly or indirectly associated with decreased BMD, better visualization of bone with low density is of significant importance in this situation. As reported in 7, 8, the CNRs of imaging low density bone (50 mg/cc), when additional filtration applied, were increased by up to 20.29% and 16.29% for 17s and 2 min scan, respectively, compared to the condition with no additional filtrations, since the imaging noise was greatly suppressed.

Similarly, the CNRs of imaging muscle were increased by up to 24.61% and 5.31% for 17 s and 2 min scan, indicating that applying additional filtrations to the micro‐CT imaging system utilized in this study may also be beneficial for imaging muscle related disorders. Another potential clinical application of micro‐CT imaging technique is to detect and characterize breast cancer through performing a micro‐CT imaging on breast biopsy specimens.^19^, ^20^ For this clinical utilization, the application of additional filtrations may provide better low‐contrast resolution when imaging microcalcification with low density or imaging tumors with similar density (CT number) as that of muscle. Therefore, additional filtrations would be recommended under the aforementioned micro‐CT imaging conditions.

Conversely, no additional filtrations would be recommended if the soft tissue, such as adipose, is preferentially to be visualized because the CNRs of imaging adipose are continuously decreased by up to 6.86% and 11.66% with maximum filtration applied. Though the impact of applying additional filtrations on imaging bones with high density and lung seems extremely significant compared to imaging others, it may not be the decisive factor, since their CNRs with respect to water or soft‐tissue background are always sufficient.

In this study, we demonstrated a unique experimental approach to validate the accuracy and/or reliability of applying the theoretical models developed from conventional CT systems to a dedicated micro‐CT system. Comparing the experimental results with the results predicted by the theoretical models, we found that, as in the derivations of CT number for water, air, muscle, adipose, and bone, the corresponding LACs under the mean energy for each material were acquired by a second‐order polynomial fitting of the existing data. In this way, the CT number variation of certain material with known characteristic could be determined theoretically. However, establishing an accurate theoretical model of predicting and calculating the CNR is difficult because the noise level which is central for CNR determination often needs to be detected and measured by experiments rather than using mathematical calculation. Thus, it would be useful and meaningful to develop a noise evaluation model for this micro‐CT scanner using an experimental data‐driven learning approach, making it possible to predict CNR variation for various scanned materials with modified beam quality.

An important issue that may affect the theoretical evaluations of CT numbers and CNRs is the utilization of the mean energy of the acquired spectrum instead of the effective energy, which is defined as the monoenergetic beam of photons that has the same HVL as the investigating spectrum of photons. This is because acquiring the effective energy and the HVL, which are related to not only the spectrum property but also the material characteristic, for each specific material seems difficult or even impossible. Nevertheless, it might account for the observation that the CNRs derived in theory and by experiment are slightly inconsistent, especially for muscle and adipose. Another reason is that the muscle and adipose used for theoretical calculations may not have identical features, such as density and homogeneity, as for experimental investigations.^(18)^

Spectral measurement in a micro‐CT system poses great challenges due to the limited space inside the gantry. Though the photon rates in this study have been successfully reduced to an acceptable level of less than 2000 p/s through using smaller pinhole collimators (i.e., the combination of 200 μm and 400 μm), the process of spectrometer adjustment is highly reliant on the operation experience and may not be effective and reproducible each time. A possible alternative is to first measure the $90^{{}^{\circ}}$ Compton scattered photons from a given sample (e.g., polymethylmetacrylate (PMMA), polyethylene, and carbon electrode) with known property. Then, the actual spectrum incident upon the scattering sample can be reconstructed from the scattered spectrum using an energy correction and the Klein‐Nishina function.^(21)^ This indirect method effectively avoids the pileup phenomenon, saves the space, and more importantly, is easily repeatable with larger pinholes such as 1000 μm or 2000 μm. Also, previously reported spectral modeling techniques can be utilized to generate the X‐ray spectrum and to predict the impact of spectral modifications on imaging quality indicators, such as CNR discussed in this study.^(22)^ However, the specificity of the modeling for certain micro‐CT system needs to be verified by experiments before they can be applied for theoretical calculation and analysis.

## V. CONCLUSIONS

This study investigated the impact of additional spectral filtrations on image quality in a micro‐CT system. The evaluations of CT number, noise level, and CNR were performed using a water‐filled mouse phantom. The experimental results agreed well with the theoretical calculations that, with a baseline of identical entrance exposure to the imaged mouse phantom, the CNRs were degraded with improved beam quality for bone with high density and soft tissue, while those were enhanced for bone with low density, lung, and muscle. The findings in this study may provide useful references for optimizing the scanning parameters of general micro‐CT systems in future imaging applications.

## ACKNOWLEDGMENTS

This research is supported in part by National Institutes of Health (NIH), RO1 CA193378, and supported in part by a grant from the University of Oklahoma Charles and Peggy Stephenson Cancer Center funded by the Oklahoma Tobacco Settlement Endowment Trust. The authors would like to acknowledge the support of Charles and Jean Smith Chair endowment fund, as well.

## References

[1] Jiang YB , Jacobson J , Genant HK , Zhao J . Application of micro‐CT and MRI in clinical and preclinical studies of osteoporosis and related disorders, Chapter 24. In: Qin L, Gerant HK, Griffith JP, Leung KS, editors. Advanced bioimaging technologies in assessment of the quality of bone and scaffold materials: techniques and applications. Berlin Heidelberg: Springer‐Verlag; 2007.

[2] Paulus MJ , Gleason SS , Kennel SJ , Hunsicker PR , Johnson DK . High resolution X‐ray Computed tomography: an emerging tool for small animal cancer research. Neoplasia. 2000;2(1‐2):62–70. 10933069 10.1038/sj.neo.7900069 PMC1531867

[3] De Clerck NM , Meurrens K , Weiler H , et al. High‐resolution X‐ray microtomography for the detection of lung tumors in living mice. Neoplasia. 2004;6(4):374–79. 15256059 10.1593/neo.03481 PMC1502108

[4] Silva MD , Savinainen A , Kapadia R , et al. Quantitative analysis of micro‐CT imaging and histopathological signatures of experimental arthritis in rats. Mol Imaging. 2004;3(4):312–18. 15802047 10.1162/15353500200404136

[5] Lerman A and Ritman EL . Evaluation of microvascular anatomy by micro‐CT. Herz. 1999;24(7):531–33. 10609159 10.1007/BF03044224

[6] Badea CT , Johnston SM , Subashi E , Qi Y , Hedlund LW , Johnson GA . Lung perfusion imaging in small animals using 4D micro‐CT at heartbeat temporal resolution. Med Phys. 2010;37(1):54–62. 20175466 10.1118/1.3264619 PMC2801733

[7] Kiessling F , Greschus S , Lichy MP , et al. Volumetric computed tomography (VCT): a new technology for noninvasive, high‐resolution monitoring of tumor angiogenesis. Nat Med. 2004;10(10):1133–38. 15361864 10.1038/nm1101

[8] Hupfer M , Kolditz D , Nowak T , Eisa F , Brauweiler R , Kalender WA , Dosimetry concepts for scanner quality assurance and tissue dose assessment in micro‐CT. Med Phys. 2012;39(2):658–70. 22320775 10.1118/1.3675400

[9] Du LY , Umoh J , Nikolov HN , Pollmann SI , Lee TY , Holdsworth DW . A quality assurance phantom for the performance evaluation of volumetric micro‐CT systems. Phys Med Biol. 2007;52(23):7087–108. 18029995 10.1088/0031-9155/52/23/021

[10] Bretin F , Warnock G , luxen A , Plenevaux A , Seret A , Bahri MA . Performance evaluation and X‐ray dose quantification for various scanning protocols of the GE eXplore 120 micro‐CT. IEEE Nucl Sci. 2013;60(5):3235–41.

[11] Boone JM , Velazquez O , Cherry SR . Small‐animal X‐ray dose from micro‐CT. Mol Imaging. 2004;3(3):149–58. 15530250 10.1162/15353500200404118

[12] Ay MR , Ahmadian A , Maleki A , Ghadiri H. Ghafarian P , Zaidi H . The influence of X‐ray spectra filtration on image quality and patient dose in the GE VCT 64‐slice Cardiac CT scanner. 3rd International Conference on Bioinformatics and Biomedical Engineering 2009. Piscataway, NJ: IEEE; 2009.

[13] Lee SC , Kim HK , Chun IK , Cho MH , Lee SY , Cho MH . A flat‐panel detector based micro‐CT system: performance evaluation for small‐animal imaging. Phys Med Biol. 2003;48(24):4173–85. 14727760 10.1088/0031-9155/48/24/014

[14] Park JY , Lee SK , Kim JY , Je KH , Schellingerhout D , Kim DE . A new micro‐computed tomography‐based high‐resolution blood‐brain barrier imaging technique to study ischemic stroke. Stroke. 2014;45(2):2480–84. 25013021 10.1161/STROKEAHA.114.006297

[15] Zhang D , Li X , Liu B . X‐ray spectral measurements for tungsten‐anode from 20 to 49 kVp on a digital breast tomosynthesis system. Med Phys. 2012;39(6):3493–500. 22755729 10.1118/1.4719958

[16] Bushberg J , Seibert J , Leidholdt M Jr , Boone J . The essential physics of medical imaging, 3rd edition Philadelphia, PA: Lippincott Williams & Wilkins; 2012.

[17] Firbank MJ , Coulthard A , Harrison RM , Williams ED . A comparison of two methods for measuring the signal to noise ratio on MR images. Phys Med Biol. 1999;44(12):N261–N264. 10616158 10.1088/0031-9155/44/12/403

[18] Hubbell JH and Seltzer SM . Tables of x‐ray mass attenuation coefficients and mass energy absorption coefficients 1 keV to 20 MeV for elements Z = 1 to 92 and 48 additional substances of dosimetric interest. National Institute of Standards and Technology Report No. 5632. Gaithersburg, MD: NIST; 1996.

[19] Tang R , Buckley JM , Fernandez L , et al. Micro‐computed tomography (Micro‐CT): a novel approach for intraoperative breast cancer specimen imaging. Breast Cancer Res Treat. 2013;139(2):311–16. 23670129 10.1007/s10549-013-2554-6

[20] Willekens I , Van de Casteele E , Buls N , et al. High‐resolution 3D micro‐CT imaging of breast microcalcifications: a preliminary analysis. BMC Cancer. 2014;14:9. 24393444 10.1186/1471-2407-14-9 PMC3893600

[21] Maeda K , Matsumoto M , Taniguchi A . Compton‐scattering measurement of diagnostic X‐ray spectrum using high‐resolution Schottky CdTe detector. Med Phys. 2005;32(6):1542–47. 16013712 10.1118/1.1921647

[22] Boone JM and Seibert JA . An accurate method for computer‐generating tungsten anode X‐ray spectra from 30 to 140 kV. Med Phys. 1997;24(11):1661–70. 9394272 10.1118/1.597953

